# Takinib Inhibits Inflammation in Human Rheumatoid Arthritis Synovial Fibroblasts by Targeting the Janus Kinase-Signal Transducer and Activator of Transcription 3 (JAK/STAT3) Pathway

**DOI:** 10.3390/ijms222212580

**Published:** 2021-11-22

**Authors:** Paul M. Panipinto, Anil K. Singh, Farheen S. Shaikh, Ruby J. Siegel, Mukesh Chourasia, Salahuddin Ahmed

**Affiliations:** 1 Department of Pharmaceutical Sciences, Washington State University College of Pharmacy and Pharmaceutical Sciences, Spokane, WA 99202, USA; paul.panipinto@wsu.edu (P.M.P.); anil.singh@wsu.edu (A.K.S.); farheen.shaikh@wsu.edu (F.S.S.); ruby.siegel@wsu.edu (R.J.S.); 2 Center for Computational Biology and Bioinformatics, Amity Institute of Biotechnology, Amity University Uttar Pradesh, Noida 201301, India; mchourasia@gmail.com; 3 Division of Rheumatology, University of Washington School of Medicine, Seattle, WA 98109, USA

**Keywords:** takinib, TAK1, rheumatoid arthritis, RASFs, THP-1, MAPK, NF-kB, JAK/STAT

## Abstract

TGF β-activated kinase 1 (TAK1) is an important participant in inflammatory pathogenesis for diseases such as rheumatoid arthritis (RA) and gouty arthritis. The central position it occupies between the mitogen activated protein kinase (MAPK) and nuclear factor kappa B (NF-κB) pathways makes it an attractive therapeutic target. As this field has developed in recent years, several novel inhibitors have been presented as having specific activity that reduces the TAK1 function either covalently as in the case of 5Z-7-oxozeanol (5Z7O) or reversibly (NG-25). However, the mechanism through which takinib elicits its anti-inflammatory activity remains elusive. While this inhibitor shows great promise, a thorough analysis of its inhibitor function and its potential off-target effects is necessary before addressing its clinical potential or its use in inflammatory conditions. An analysis through Western blot showed an unexpected increase in IL-1β-induced TAK1 phosphorylation—a prerequisite for and indicator of its functional potential—by takinib while simultaneously demonstrating the inhibition of the JAK/STAT pathway in human rheumatoid arthritis synovial fibroblasts (RASFs) in vitro. In THP-1 monocyte-derived macrophages, takinib again led to the lipopolysaccharide-induced phosphorylation of TAK1 without a marked inhibition of the TAK1 downstream effectors, namely, of c-Jun N-terminal kinase (JNK), phospho-c-Jun, NF-κB phospho-p65 or phospho-IκBα. Taken together, these findings indicate that takinib inhibits inflammation in these cells by targeting multiple signaling pathways, most notably the JAK/STAT pathway in human RASFs.

## 1. Introduction

Rheumatoid arthritis is an autoimmune disease with complex etiology that leads to progressive pain, inflammation, and eventual joint failure [1]. Major contributions to RA pathogenesis are made by activated synovial fibroblasts (RASFs), which produce inflammatory mediators and recruit inflammatory immune cells while also destroying cartilage via invasion and matrix metalloproteinase (MMP) production [2,3]. Invasive RASFs further propagate RA pathogenesis via the production of interleukin-6 (IL-6), which facilitates bone resorption and prevents bone production [4,5,6]. Our understanding of the etiology and pathology of this inflammatory condition has developed dramatically, however, safe and effective therapy remains elusive.

While traditional therapeutics for RA comprise of disease modifying anti-rheumatic drugs (DMARDs), the introduction of cytokine-targeted biologics as treatments introduced the potential for novel, more efficacious therapy. The success of tocilizumab (an anti-IL-6R antibody) and tofacitinib as an inhibitor of JAK/STAT led to the successful use of JAK/STAT targeted therapy in clinics for RA [7,8]. Its clinical usage was short-lived, however, as post-market surveillance revealed increased vulnerability to opportunistic infections, cardiovascular and thromboembolic events [9,10,11]. The continuing search for a therapeutic target with an acceptable safety profile has shifted to novel targets. TAK1 has emerged as a prominent therapeutic target and is proximal to the IL-1β, Tumor Necrosis Factor-alpha (TNFα) and Toll-like (TLR) receptors and the upstream of both the cytokine-producing nuclear factor kappa-B (NF-κB) and mitogen activated protein kinase (MAPK) pathways [11,12,13,14,15]. The expectation is that the inhibition of both NF-κB and MAPK will lead to reduced inflammation via inhibited cytokine production. Mutational studies have uncovered substantial mechanistic insights into TAK1’s function, including the importance of phosphorylation of Thr^184/187^ in the kinase activation loop [16,17,18].

Several TAK1 inhibitors are now in preclinical use, including the covalent type-1 inhibitor 5Z-7-oxozeanol (5Z7O) and the DFG-out conformation reversable type-2 inhibitor NG-25, for which mechanisms of action are well established [19,20]. A third inhibitor, takinib, has been shown to reduce proinflammatory mediators and is proposed to act as a specific inhibitor of TAK1 [21,22] although its mechanism have not yet been completely resolved. To better understand the signaling mechanisms through which takinib exhibits its anti-inflammatory activity, we tested the effect of takinib on IL-1β-activated human RASFs as well as on IL-6-activated RASFs in vitro. Additionally, we used lipopolysaccharide (LPS)-activated THP-1 monocyte-derived macrophages to determine the effect of takinib on TLR4-induced inflammatory signaling. In this study, we aimed to decipher the underlying mechanism of action of takinib and identified the JAK/STAT pathway as a potential target of takinib in IL-1β-activated human RASFs in vitro. These findings suggest that takinib acts in a non-specific manner that varies by cell and stimulation. 

## 2. Results

### 2.1. Inhibition of TAK1 Reduces Proinflammatory Mediators Secreted by RASFs and THP-1 Monocyte-Derived Macrophages

**Figure 1:**
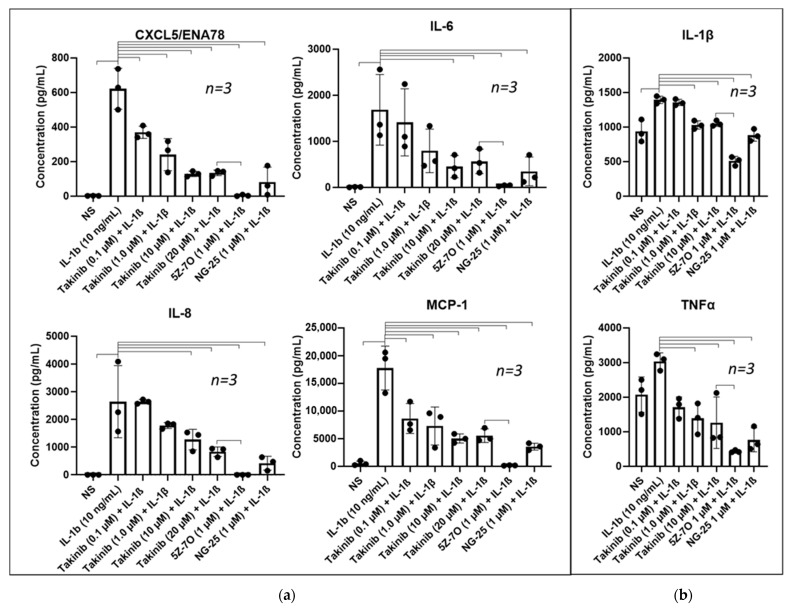
Inhibition of TAK1 reduces proinflammatory mediators. (**a**) RASFs were pre-treated with selected inhibitors followed by 24 h stimulation with IL-1β (10 ng/mL). Supernatants were collected and analyzed by ELISA. Takinib demonstrates inhibition of CXCL5, IL-6, IL-8, and MCP-1 though not as completely as 5Z7O or NG-25. (**b**) THP-1 monocyte-derived macrophages pre-treated with selected inhibitors followed by 24 h stimulation with LPS (10 µg/mL). Supernatants collected and analyzed by ELISA demonstrate significant reduction in both IL-1β and TNFα. 5Z7O lowered both cytokines significantly more than any treatment of takinib. Significance bars represent *p* < 0.05 in one-way ANOVA with Dunnett’s post hoc test for multiple comparisons to either IL-1β or LPS.

We began this study with a comparison of known TAK1 inhibitors takinib, 5Z7O, and NG-25 to investigate their relative inhibition of pro-inflammatory cytokines and chemokines. Human RASFs were serum-starved overnight, pre-treated for two hours with TAK1 inhibitors followed by a 24 h activation period with recombinant human IL-1β (10 ng/mL). The conditioned media was analyzed for secreted proteins by ELISA. Takinib (0.1–20 µM) displayed significant dose-dependent decreases in IL-1β-activated ENA-78/CXCL5, IL-6, and MCP-1/CCL2 production (Figure 1a). IL-8 demonstrated a downward trend and reached statistical significance at a 10 µM takinib dosing in a one-way ANOVA comparison to the IL-1β stimulated samples (Figure 1a). In comparison to takinib, 1 µM 5Z7O displayed statistically significant reductions compared to both IL-1β stimulated samples. Treatment with 1 µM NG-25 significantly lowered all four pro-inflammatory mediators in all cases. In THP-1 monocyte-derived macrophages (Figure 1b), 1 µM Takinib reduced IL-1β production by an average of 26% across the replicates compared to LPS treated (10 µg/mL) samples, while 1 µM of 5Z7O or NG-25 reduced LPS-induced IL-1β supernatant concentrations by 63% and 36%, respectively (Figure 1b). Takinib demonstrated a strong inhibition of TNFα at even 0.1 µM concentrations (−54%), though 5Z7O and NG-25 treatments reduced LPS-induced TNFα production by a significantly higher degree (−86% and −75%, respectively). These results demonstrate that takinib does indeed have significant anti-inflammatory ability, though inhibition is less effective than for the known inhibitors of TAK1. Additionally, our MTT-based cell viability results revealed takinib and 5Z7O to be cytotoxic at 24 h at the 10 and 1 µM concentrations, respectively (Supplementary Figure S1) suggesting that the reduction of takinib at higher concentrations may be partially attributed to the loss of cell viability.

### 2.2. Takinib Inhibits STAT3 and JNK Phosphorylation in IL-1β, but Not IL-6 Stimulated Human RASFs

**Figure 2:**
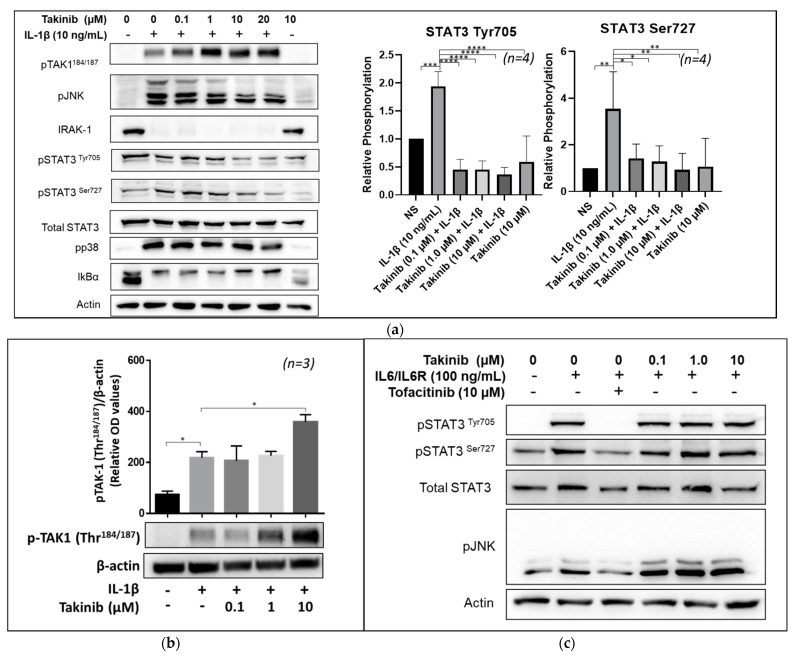
Treatment of RASFs with takinib and either IL-1β or IL-6 trans-signaling demonstrates differential activation of signaling pathways. (**a**) RASFs were pre-treated for two hours with takinib followed by 30 min stimulation with IL-1β (10 ng/mL). Whole cell extracts were collected and analyzed by Western Blot. Takinib induces phosphorylation of TAK1 while reducing STAT3 phosphorylation at the Tyr705 and Ser727 residues in *n* = 4 RASF cell lines. (**b**) Takinib demonstrates increasing phosphorylation of the Thr184/187 residues in the kinase loop of TAK1. (**c**) RASFs were pre-treated with takinib followed by 30 min stimulation with IL-6 and IL-6R (100 ng/mL). Whole cell extracts analyzed by Western Blot show no effect on STAT3 phosphorylation by Takinib in *n* = 3 RASF lines. * = *p* < 0.05, ** = *p* < 0.001, *** = *p* < 0.0001, **** = *p* < 0.00001.

Next, we investigated the effect of takinib on the signaling capability of major inflammatory cytokine pathways in human RASFs. Overnight-starved human RASFs were pre-treated as described above with a dose range of takinib (0.1–20 µM) followed with 30 min IL-1β stimulation. Whole-cell extracts were analyzed by Western Blotting. Surprisingly, takinib induced dose-dependent phosphorylation of TAK1^Thr184/187^ in IL-1β-treated samples while also demonstrating a significant inhibition of STAT3^Tyr705^ and STAT3^Ser727^ phosphorylation (Figure 2a,b). As expected, JNK phosphorylation decreased with an increasing takinib concentration and the total STAT3 levels were unaffected. Treatment with takinib did not lead to a significant inhibition of p38 signaling and was insufficient to prevent the degradation of IRAK1 and I-κBα triggered by IL-1β. We then examined whether STAT3 inhibition functions via canonical or non-canonical STAT3 activation by stimulating human RASFs with an IL-6 and IL-6 receptor. In RASFs, IL-6 trans-signaling induced JAK/STAT3 activation was not inhibited by takinib pretreatment, whereas tofacitinib (a JAK inhibitor) demonstrated a complete inhibition of JAK/STAT signaling (Figure 2c). Additionally, takinib was unable to inhibit the JNK signaling pathway as would normally be expected from upstream TAK1 kinase inhibition. 

### 2.3. Takinib Inhibits STAT3 Nuclear Translocation and DNA-Binding Activity in Human RASFs

**Figure 3:**
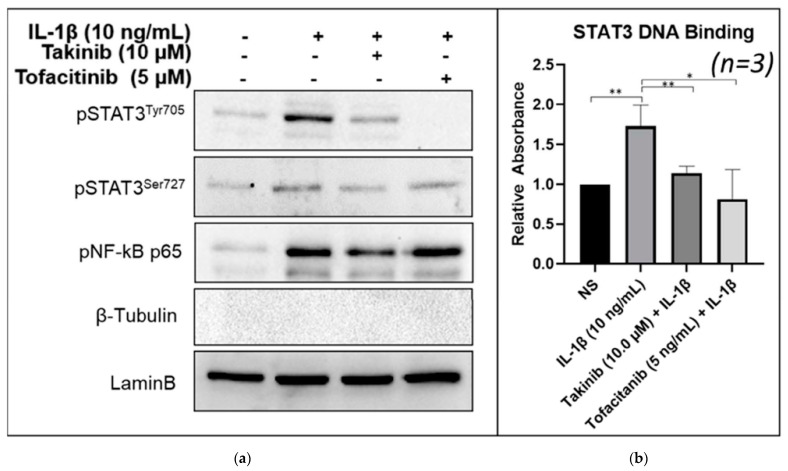
Treatment of RASFs with takinib or Tofacitinib and IL-1β reduces nuclear phospho-STAT3^Tyr705^ and STAT3’s DNA-binding ability. (**a**) RASFs were pre-treated with takinib or tofacitinib followed by 30 min stimulation with IL-1β (10 ng/mL). Purified nuclear extracts from *n* = 3 cell lines were collected and analyzed by Western Blot. In *n* = 3 RASF cell lines. (**b**) RASFs were pre-treated with takinib followed by 30 min stimulation with IL-1β (10 ng/mL). Nuclear extracts analyzed by ELISA show reduced DNA binding activity in *n* = 3 RASF cell lines treated with takinib and tofacitinib. * = *p* < 0.05, ** = *p* < 0.001.

To further examine the impact of STAT3 phosphorylation inhibition by takinib, we isolated the nuclear extract from IL-1β stimulated human RASFs, with or without takinib pre-treatment. A Western blot analysis of the purified nuclear extract from RASFs revealed markedly reduced levels of nuclear pSTAT3^Tyr705^, but not of pSTAT3^Ser727^ in IL-1β-stimulated samples treated with either 10 µM takinib or 5 µM tofacitinib (Figure 3a). Surprisingly, takinib (10 µM) was not potent enough to inhibit IL-1β-induced nuclear translocation of NF-κBp65, suggesting that STAT3 and JNK pathways are the primary target of takinib in IL-1β-activated signaling in human RASFs (Figure 3a).

We hypothesized that this reduction in Tyr705 phosphorylation may interfere with the ability of STAT3 to act as a transcription factor by reducing its ability to bind to DNA. To test this hypothesis, we used a purified nuclear extract from human RASFs from the above-mentioned treatment for determining the DNA-binding activity. Results from the DNA binding ELISA of nuclear extracts showed that takinib significantly inhibited IL-1β-induced STAT3 DNA binding activity, which was comparable to the inhibitory potential of the JAK inhibitor tofacitinib in IL-1β treated samples (Figure 3b).

### 2.4. Takinib Phosphorylates TAK1 and Fails to Inhibit NF-κB or MAPK Signaling in LPS-Stimulated THP-1 Macrophages

**Figure 4:**
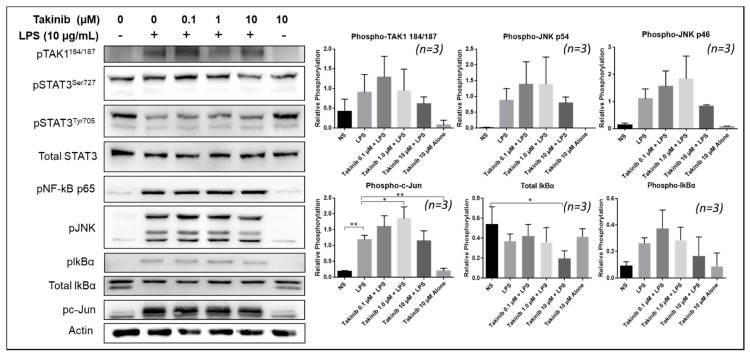
Takinib phosphorylates TAK1 and fails to inhibit the MAPK/NF-kB pathways in THP-1 macrophages. THP-1 monocytes were treated with 100 ng/mL PMA for 3 h, left to rest in fresh RPMI with 10% FBS overnight, followed by 2 h treatment with takinib and an additional 30 min LPS (10 µg/mL) stimulation. NF-kB p65, IkBα, JNK, and c-Jun phosphorylation were all uninhibited, while TAK1 phosphorylation increased under LPS and takinib treatment. * = *p* < 0.05, ** = *p* < 0.001.

Next, we examined the effect of takinib treatment on TLR4-induced TAK1 signaling. Using phorbol 12-myristate 13-acetate (PMA) treated THP-1 monocytes, pre-treated with takinib and stimulated for 30 min with 10 µg/mL LPS, we isolated the whole-cell extract and performed Western Blotting. Pretreatment with takinib further increased LPS-induced TAK1 phosphorylation at Thr^184/187^, with a modest inhibitory effect on Ser^727^ phosphorylation site (Figure 4). However, takinib did not lead to an inhibitory effect on LPS-induced pNF-κBp65 and led to a potential increase in pJNKp46/p54 (Figure 4). Additionally, a densitometric analysis of Western blots showed no significant inhibition of pJNKp46/p54 or its downstream substrate p-c-Jun. Finally, STAT3 signaling in LPS-stimulated cells was unaffected by treatment with takinib. 

### 2.5. Docking Simulations Using Takinib and STAT3 Demonstrate a Potential Site of Interaction

**Figure 5:**
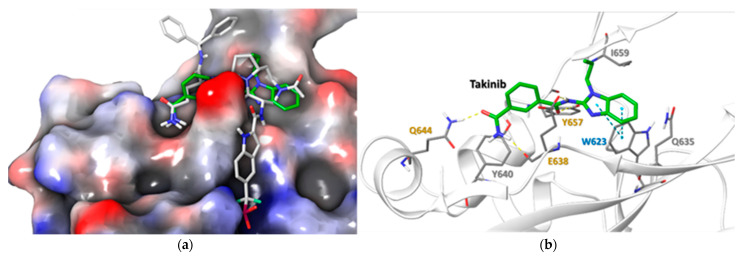
Docked pose of takinib in the binding site of STAT3 (PDB ID: 6NJS): (**a**) Showing electrostatic potential surface of the STAT3 with co-crystallized ligand (SD-36) and docked takinib. The red and blue color depict electronegative and electropositive surfaces while white color denotes hydrophobic surface. (**b**) The interaction of takinib with the binding site residues of STAT3. The light grey labelling represents the residues that leads to hydrophobic interaction with the ligand. The yellow and blue dotted line represent H-bonding and π-π interaction, respectively. The ligand in the active site is represented by thick stick model while interacting residues are represented in thin stick model.

The electrostatic potential surface of STAT3 shows that the ligand binding cavity of STAT3 is mainly lined by hydrophobic residues (Figure 5a). The binding site of STAT3 is shallow and wide. The docked takinib aligned well with the orientation of the core of the co-crystallized ligand (SD-36) in the binding cavity and presented similar active site binding partners [22]. Both STAT3 and takinib demonstrated a binding energy of −6.610 Kcal/mol. The H-boding and hydrophobic interactions contribute to the binding. The E638, Q644 and Y657 residues form the network of the H-bond with the ligand, while W623, Y640, Y657 and I659 residues presented hydrophobic interaction with the ligands. Their similarities are further observed in the π-π interaction between W623 and the benzimidazole ring of takinib (Figure 5b). A list of potential interacting residues modeled using Schrodinger suit 2020.3 [23] and their impact on STAT3 functions can be found in Table 1.

## 3. Discussion

The position of TAK1 at the nexus of the MAPK and NF-kB pathways makes it an appealing target for anti-inflammatory therapy. This study demonstrates that although takinib is an effective inhibitor of inflammation, its mode of action is different from the previously described target, TAK1. Our findings demonstrate that takinib preferentially inhibited IL-1β-induced STAT3 phosphorylation, nuclear translocation, and DNA-binding in human RASFs in vitro. Furthermore, while takinib displayed a modest inhibition of JNK pathway in IL-1β stimulated RASFs, it failed to replicate this inhibition of inflammation caused by canonical IL-6 trans-signaling in human RASFs, or in LPS stimulated THP-1 macrophages. Importantly, takinib induced the phosphorylation of TAK1 at Thr^184/187^ residues, which are important kinase domain and ATP-binding sites of TAK1, in both human RASFs and THP-1 macrophages. Molecular docking studies suggest that takinib interacts with some of the key residues that other STAT3 inhibitors are known to require. In our study, we found that the participation of Y640, Y675, I659 provides a hydrophobic pocket in which E638, Q644 and W623 orient and stabilize takinib, relative to STAT3, which is consistent with previously published reports of other STAT3 inhibitors. Together, these findings provide evidence that takinib may target signaling proteins that do not crosstalk with the TAK1/MAPK pathway and can inhibit inflammatory mediators via the STAT3 pathway.

The inflammatory milieu and heterogenous nature of RA pathogenesis lends itself to complex overlapping cell signaling mechanisms and a difficulty in targeting a specific kinase or protein for therapeutic purposes. While the roles of many inflammatory mediators are well known, questions remain with respect to the inflammatory hierarchy and the best approach for safe, efficacious therapy [28]. This complexity makes the goal of one-drug, one-target therapy increasingly difficult and requires pharmacologists in the field to be cognizant of the importance of inflammatory cytokines in disease pathogenesis. The limitations of currently used biologics that target proinflammatory cytokines, such as TNFα and IL-6R, have led to the search for small molecule inhibitors that are able to target common signaling kinases, which are central to mediating inflammatory signals, with TAK1 emerging as one promising therapeutic target for RA [12,15]. Two specific classes of validated TAK1 inhibitors (a covalent-binding irreversible type I (5Z7O) and a DGF-out conformation based reversible type II (NG-25)) have provided opportunities for preclinical testing. Takinib was shown to competitively inhibit TAK1 ATP-binding in DFG-in conformation to enhance TNFα-induced apoptosis in RASFs [21] and to ameliorate collagen-induced arthritis (CIA) in mice [29,30], however, its effect on TAK1 kinase activity in these cells has yet to be validated. Importantly, although TAK1 is activated by TNFα [31], it does not exclusively rely on association with TRAF2 to form an activation complex [32], which allows for the bifurcation of cell signaling to MAPK and NF-κB pathways at the TAK1/TAB1 complex, meaning that TAK1 becomes dispensable in TNFα-driven signaling. 

IL-1β plays an important role in synovial inflammation and bone/cartilage destruction in RA [33,34]. Even in TNFα-driven arthritic human TNF-transgenic (hTNF-tg) mice, IL-1 inhibition completely abolished cartilage and bone destruction, suggesting it to be a crucial mediator of RA pathogenesis [35,36,37]. Our findings provide evidence that, in human RASFs, takinib inhibits inflammatory markers mainly by interfering with IL-1β-induced STAT3 and JNK, which suggests that takinib does not directly effect TAK1, but instead effects multiple pathways in RASFs to induce its anti-inflammatory activity. Furthermore, we evidence, using in silico docking studies, that sites at which takinib interact with STAT3 residues are important for confirmational changes, consequential phosphorylation or activation, and in nuclear translocation for DNA binding activity. This was attested by our findings in human RASFs that indeed takinib inhibits IL-1β-induced nuclear translocation and in DNA binding activity of STAT3. These finding suggest that the anti-inflammatory activity of takinib is mediated by multiple pathways in RASFs and THP-1 macrophages, that are independent of TAK1 in the signaling hierarchy of these cells. Additionally, the current findings, for which takinib maintained TAK1 phosphorylation at Thr^184/187^ in RASFs and THP-1 macrophages via IL-1β or LPS stimulation, respectively, and inhibited p-JNK expression, suggest that takinib may have a more profound effect on JNK kinase rather than on TAK1 in its mechanisms of suppressing inflammation.

In the study by Totzke et al. [21] the treatment of human RASFs with takinib induced apoptosis and reduced IL-6 production. In a follow-up study by Scarneo et al., the authors provided evidence that takinib, at a similar dose range, has no adverse effect on RASF viability, but significantly inhibited chemokine production by TNFα-activated human RASFs. However, the mechanisms through which takinib relates to the inhibition of TAK1 kinase in these cells remains elusive. Furthermore, the activation of TNFα-induced apoptosis signaling kinase 1 (ASK1) immediately after TRAF2 activation, branches signaling to the JNK pathway that contributes to programmed cell death or to apoptosis, as observed in case of takinib. Our findings, which elucidate the role of STAT3, bridges the gap in understanding of the pharmacological action of takinib by identifying a primary target of takinib in human RASFs. While these results are open to different interpretations, the available literature detailing the role of STAT3 in LPS-induced TNFα and other forms of chemokine production in macrophage cells [38,39] further attest to our argument that takinib elicits its anti-inflammatory effects primarily through suppression of JAK/STAT pathway, not TAK1 driven signaling, in human RASFs and possibly macrophages.

In conclusion, this is the first study to test the effect of takinib in IL-1β stimulated inflammation primary human RASFs and to provide molecular insights into the possible mechanism of its anti-inflammatory activity in vitro. Our findings revealed that the pharmacological action of takinib in reducing cytokine production may not be due to its inhibition of TAK1, but of other signaling proteins including STAT3 in these cells. Additionally, the increased phosphorylation of TAK1 and the lack in inhibition of the MAPK and NF-kB pathways in macrophages raise further questions about the selectivity of takinib in instances of IL-1β-driven diseases. These findings warrant further investigation before takinib is validated as a selective TAK1 inhibitor, by testing its therapeutic potential.

## 4. Materials and Methods

### 4.1. Antibodies and Reagents

Recombinant IL-1β were purchased from R&D Systems (Minneapolis, MN). Antibodies for p-TAK1^(Thr184/187)^, TAK1^(Ser412)^, p-IRAK4^(Thr345/Ser346)^, p-IRAK1^(Thr209)^, p-TAB2^(Ser372)^, IL-1β, p-STAT3^(Tyr705)^, p-STAT3^(Ser727)^, p-SAPK/JNK^(Thr183/Tyr185)^, and IκBα were purchased from Cell Signaling Technologies (Danvers, MA; Cat# 90C7, 9339S, D6D7, T209, 8155, D3U3E, 9145S, 9136S, 4671S and 4812S). β-Actin and IRAK1 were purchased from Santa Cruz Biotechnology (Santa Cruz, CA, USA; sc-47778; H273). Lipopolysaccharide (LPS), takinib and Tofacitinib were purchased from Sigma (St. Louis, MO, USA). The STAT3 Transcription Factor Assay Kit (Cat: 601950), 5Z-7-oxozeaenol, and NF-κB (p65) Transcription Factor Assay Kit (Cat: 10007889) were purchased from Cayman Chemicals (Ann Arbor, MI, USA) and NG 25 trihydrochloride was purchased from Axon Medchem (Reston, VA, USA). 

### 4.2. Culturing of Human RASFs and THP1

De-identified human RA synovium tissues were obtained from Cooperative Human Tissue Network (CTHN; Columbus, OH, USA) and National Disease Research Interchange (NDRI; Philadelphia, PA USA). Normal and RA tissues were obtained from total joint replacement surgery or synovectomy under an Institutional Review Board (WSU-IRB) approved protocol, in compliance with the Helsinki Declaration. The donor population included both males and females diagnosed with RA whereby the average age of the donors was 57 ± 27 years. RA tissues were digested in collagenase, before being seeded in 72 cm^2^ flasks. Cells were grown in an RPMI 1640 medium supplemented with 15% fetal bovine serum (FBS), 5000 U/mL penicillin, 5 mg/mL streptomycin, and 10 µg/mL gentamicin. Upon confluency (>85%), cells were passaged with brief trypsinization. The experiments were performed using cells that were passed at least 4 to 5 times to ensure pure fibroblast population. For experimental purposes, RASFs between passages 5–10 were used. All treatments were performed in serum free media. All experiments were performed on at least 3–4 cell lines established from different RA donors for this study. Human monocytic leukemia (THP-1) cells were purchased from ATCC (88081201-1VL, Manassas, VA, USA) and maintained in an RPMI 1640 culture media with 10% fetal bovine serum (FBS) and antibiotics. For the experiments, THP-1 cells were differentiated to a macrophage population with phorbol 12-myristate 13-acetate (100 ng/mL) for 3 h, followed by media replacement with fresh Opti-MEM media overnight.

### 4.3. Treatment of RASFs and THP-1

RASFs were seeded in 6-well plates or 100 mm dishes, grown to >85% confluency. Cells were placed in serum-free media overnight prior to treatments. To study inhibitors, cells were pre-incubated with takinib (0.1–20 µM), 5Z-7-oxozeaenol (1 µM), NG 25 trihydrochloride (1 µM) for 2 h prior to IL-1β (10 ng/mL) stimulation for 30 min to study changes in signaling, or for 24 h, to evaluate the production of IL-6, IL-8, CXCL5 and MCP-1. Likewise, overnight-differentiated THP-1 cells were pre-incubated with takinib (0.1–20 µM), 5Z-7-oxozeaenol (1 µM), NG 25 trihydrochloride (1 µM) for 2 h prior to stimulation from LPS (10 ng/mL) for 30 min or 24 h and subjected to Western blotting or ELISA.

### 4.4. Assay for Cytokine Production

The conditioned media from RASFs or THP-1 was collected from 24 h IL-1β- or LPS-stimulated samples which were spun down at 10,000 rpm for 10 min at 4 °C to remove particulate matter and where then collected in fresh Eppendorf tubes. The collected supernatants were analyzed for human IL-1β, TNFα, ENA-78/CXCL5, MCP-1/CCL2, IL-6 and IL-8 levels using colorimetric sandwich ELISA kits (R&D Systems, Minneapolis, MN, USA) as per the manufacturer’s instructions.

### 4.5. Western Immunoblotting

The whole-cell extract was prepared using RIPA buffer (50 mM Tris pH 7.6, 150 mM NaCl, 1% Triton X-100, 1 mM EDTA, 1 mM DTT, 0.5% sodium deoxycholate, and 0.1% SDS) containing protease and phosphatase inhibitors (Roche, Basel, Switzerland). Protein content was measured using Bio-Rad DC method (Bio Rad, Hercules, CA, USA). An equal amount of protein (25 µg) was loaded for each sample and separated on acrylamide gel before being transferred to a PVDF membrane (EMD Millipore, Billerica, MA, USA). Blots were then blocked in TBST containing 5% nonfat dry milk for two hours prior to overnight incubation with the respective primary antibody (see section on antibodies and reagents) with dilution according to the manufacturer’s instructions. Protein bands were visualized using chemiluminescence and analyzed using Image Lab software (Bio-Rad) for band intensity. Blots were probed with β-actin to ensure equal loading. Relative STAT3 phosphorylation was determined by normalizing pSTAT3 and total STAT3 bands with β-actin, using a ratio of pSTAT3/Total STAT3. We performed pTAK1 phosphorylation using a ratio of pTAK1/β-actin.

### 4.6. Cell Fractionation

The fractionation method of Zhu et al. was modified accordingly [12,14]. RASFs were grown in 100 mm plates with a confluency of up to 90%, pretreated with takinib, tofacitinib or untreated, followed by IL-1β (10 ng/mL) stimulation for 24 h. Afterwards, cells were washed once in cold 1X PBS and lysed with 1 mL of Cytoplasmic lysis buffer (10 mM Tris-HCL, pH 7.9, 0.34 M Sucrose, 3 mM CaCl_2_, 2 mM magnesium acetate, 0.1 mM EDTA, 1 mM DTT, 0.5% CA630 and protease inhibitors). The cell pellet that formed was gently resuspended using a wide mouth tip, followed by incubation on ice for 30 min. Nuclei were pelleted by centrifugation at 3500× *g* for 15 min at 4 °C. Following this, centrifuge cytoplasmic extract was stored in a pre-chilled 1.5 mL Eppendorf tube.

The remaining nuclei pellet was washed with 1 mL of Cytoplasmic lysis wash buffer as before, followed by lysis in 0.2 mL of RIPA buffer (50 mM Tris pH 7.6, 150 mM NaCl, 1% Triton X-100, 1 mM EDTA, 1 mM DTT, 0.5% sodium deoxycholate, and 0.1% SDS) and kept on ice for 30 min. The nuclear extract was collected via centrifugation at 15,000× *g* for 30 min at 4 °C in a fresh Eppendorf tube.

### 4.7. DNA Binding Assay for STAT3

Five µg of nuclear extract was used for DNA binding activity, to form unstimulated and IL-1β- (10 ng/mL) stimulated samples with or without takinib and Tofacitinib treatments for 2 h at room temperature for binding as per the manufacturer’s instructions.

### 4.8. Molecular Dynamics (MD) Simulation Studies

To identify potential interactions of takinib with STAT3 in silico, the 3D structures of human STAT3 (PDB ID: 6NJS) [22] were prepared using protein preparation wizard. All docking calculations were performed using Schrodinger suit 2020.3 [23]. The protonation states of all the titratable residues were assigned at a physiological pH using PROPKA. Retrained minimization was performed using 0.30 Å root-mean-square deviation (RMSD) via optimized potentials for the liquid simulation’s extended (OPLS3e) force field. The 30 Å grid was generated around the co-crystallized small molecule (SD-36). The takinib structure, used for docking, was prepared at pH 7.0 ± 2.0 using LigPrep module. The docking calculations were performed using GLIDE module. The takinib molecule was first submitted for Standard Precision (SP) docking to generate 10 docking poses. These poses were then submitted to Extra Precision (XP) docking. The selection of the more suitable pose was made on the basis of the energy and interaction of takinib with the active site residues. To better understand the interactions between the active site and takinib, these poses were then submitted for inducedFit docking. In induced fit docking, we maintained the flexibility of active site residues and takinib so that they were able to adjust themselves to a better binding pose and affinity.

## Figures and Tables

**Table 1:** Predicted takinib/STAT3 Binding Interactions and Known STAT3 Residue Function.

Residue	Takinib Interaction	Function
E638	H-Bond	Amide H-Bond works with Q644 to stabilize ligand [24]
Q644	H-Bond	Stabilizes side chain H-bonds [25]
Y640	Hydrophobic	Modulates Tyr705 phosphorylation, pocket hydrophobicity [26]
Y657	H-bond, Hydrophobic	Works with E638 & Y640 to modulate pocket hydrophobicity [22,24,26]
I659	Hydrophobic	Ligand stabilization, pocket hydrophobicity [24,26]
W623	Hydrophobic, π-π	Induces ligand conformational fit, stabilizes ligand in pocket [24,27]

## Data Availability

The data presented in this study are available on request from the corresponding author.

## Supplementary Materials

The following are available online at https://www.mdpi.com/article/10.3390/ijms222212580/s1.
