# Supplementary material for: Takinib Inhibits Inflammation in Human Rheumatoid Arthritis Synovial Fibroblasts by Targeting the Janus Kinase-Signal Transducer and Activator of Transcription 3 (JAK/STAT3) Pathway
Source: Int J Mol Sci. 2021 Nov 22;22(22):12580. doi: 10.3390/ijms222212580 (PMC8621335; doi:10.3390/ijms222212580)
Supplement: Supplementary file 1 [file ijms-22-12580-s001.zip › ijms-1422834-supplementary.pdf]

Supplementary Materials

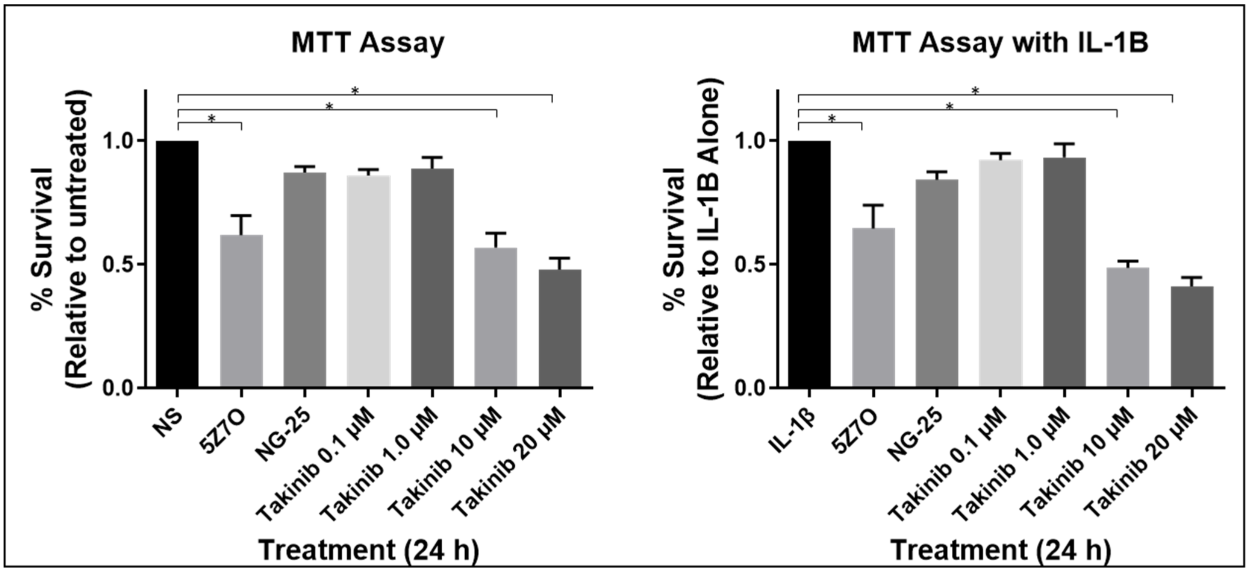

Supplementary Figure S1: Treatment with takinib and 5Z7O cause cell death. RASFs were starved overnight then treated with either 5Z7O, NG-25, or various does of takinib with and without IL-1β (10ng/ml) for 24 hours. Takinib caused significant cell death beginning at 10 μM. MTT assay was performed on n=3 RASF cell lines from different donors. Statistical analysis was performed using one-way ANOVA with Dunnett's post-hoc test for multiple comparisons. Significance level is $p<0.05$.
